# Evidence-informed urban health and sustainability governance in two Chinese cities

**DOI:** 10.5334/bc.90

**Published:** 2021-06-28

**Authors:** Helen Pineo, Ke Zhou, Yanlin Niu, Joanna Hale, Catherine Willan, Melanie Crane, Nici Zimmermann, Susan Michie, Qiyong Liu, Michael Davies

**Affiliations:** UCL Institute for Environmental Design and Engineering, The Bartlett School of Environment Energy and Resources, University College London (UCL), London, UK; State Key Laboratory of Infectious Disease Prevention and Control, Collaborative Innovation Center for Diagnosis and Treatment of Infectious Diseases, National Institute for Communicable Disease Control and Prevention, Chinese Center for Disease Control and Prevention, Beijing, China; Centre for Behaviour Change, University College London (UCL), London, UK; Bartlett Faculty of the Built Environment, University College London (UCL), London, UK; The Australian Prevention Partnership Centre, Sydney School of Public Health, The University of Sydney, Sydney, NSW, Australia; UCL Institute for Environmental Design and Engineering, The Bartlett School of Environment Energy and Resources, University College London (UCL), London, UK; Department of Clinical, Educational and Health Psychology, University College London (UCL), London, UK; Chinese Center for Disease Control and Prevention, Beijing, China; UCL Institute for Environmental Design and Engineering, The Bartlett School of Environment Energy and Resources, University College London (UCL), London, UK

**Keywords:** cities, environmental risks, evidence-based policy, governance, public health, sustainability, urban health, China

## Abstract

Sustainable development is best supported by intersectoral policies informed by a range of evidence and knowledge types (*e*.*g*. scientific and lay). Given China’s rapid urbanisation, scale and global importance in climate mitigation, this study investigates how evidence is perceived and used to inform urban health and sustainability policies at central and local levels. Well-informed senior professionals in government/scientific agencies (12 in Beijing and 11 in Ningbo) were interviewed. A thematic analysis is presented using deductive and inductive coding. Government agency participants described formal remits and processes determining the scope and use of evidence by different tiers of government. Academic evidence was influential when commissioned by government departments. Public opinion and economic priorities were two factors that also influenced the use or weight of evidence in policymaking. This study shows that scientific evidence produced or commissioned by government was routinely used to inform urban health and sustainability policy. Extensive and routine data collection is regularly used to inform cyclical policy processes, which improves adaptive capacity. This study contributes to knowledge on the ‘cultures of evidence use’. Environmental governance can be further improved through increased data-sharing and use of diverse knowledge types.

## 1 Introduction

Rapid urbanisation in China has coincided with increasing global public awareness of the health impacts of environmental pollution, alongside growing attention to the climate crisis. The United Nations (UN) Habitat’s New Urban Agenda has focused attention on the potentially transformative role of cities for sustainable development across global settings (Parnell 2016). Through Sustainable Development Goal (SDG) 11, cities are called upon to be ‘safe, inclusive, resilient, and sustainable’ through the provision of suitable housing and transport, whilst reducing environmental impacts and promoting community participation, among other targets (United Nations Economic and Social Council 2016). In this new urban focus, scholars have called attention to the complexity of the factors affecting sustainability and health in cities (Gatzweiler *et al*. 2018) and the potential for policy instruments that support co-benefits for social, economic and environmental outcomes (Dora *et al*. 2015). Such policy action should be intersectoral and informed by a range of knowledge. Thus, there is a body of work on how scientific evidence can be leveraged to support healthy, equitable and sustainable urban development policies.

China’s rapid industrialisation and economic growth since the early 1990s, coupled with increasing population, has resulted in greater environmental pressures and public expectations for quality of life. Urban populations more than doubled from the late 1970s to 2010 (Schneider & Mertes 2014), with growth concentrated in coastal cities due to government support (Tan *et al*. 2016). Rapid urbanisation and environmental factors are important influences on Chinese population health and liveability (Yang *et al*. 2018; Zhan *et al*. 2018). Air pollution (outdoor and indoor), water shortages and pollution, low levels of physical activity and extreme weather events are key issues linking urban sustainability and health in China. Environmental pollution is an important issue for the Chinese public (Zhang & Barr 2013) and a key challenge that must be overcome for the country to meet the SDG Agenda 2030 goals, which is important for international standing and economic development.

The study of evidence use by policymakers is emerging as a stand-alone field, while research on this topic has tended to occur in parallel across disciplines without cross-pollination of theory (Oliver & Boaz 2019). Although there is much research on how to translate research findings for policymakers (Oliver & Cairney 2019), there is a question about what types of evidence and translation methods can be applied in different policy contexts. There has been a call for further investigations of ‘cultures of decision-making and institutional evidence use’, referring to how ‘evidence is discussed, made sense of, negotiated and communicated’ alongside ‘case studies of different types of policymaking and the evidence diets consumed’ in different settings (Oliver & Boaz 2019: 4–6). An evaluation of policymakers’ (including multiple policy sectors and countries) conceptualisations of ‘evidence’ identified trials, literature reviews, needs assessments, surveys of public views or preferences, public consultation, case studies, expert opinion, routine data and statistics (Lorenc *et al*. 2014).

Theory and empirical research suggest that evidence use is not a linear process and that expert knowledge may be one of many other considerations affecting policymaking (Cairney & Oliver 2017; Oliver *et al*. 2014; Pineo *et al*. 2019). Context-specific studies have investigated the wide range of factors that influence policymakers alongside evidence, including public support for policies, pressure from other tiers of government and conflicts with other priorities (Allender *et al*. 2009; Onwujekwe *et al*. 2015; Pineo *et al*. 2020; Xufeng 2013). A general consensus is that when evidence is used, it meets the specific needs of policymakers at a point in time. Scientists and policy actors who develop trusted relationships over time can influence each other in terms of the setting of research agendas and provision of timely policy-relevant information.

The use of research evidence in China may differ from other countries given its high expenditure on research and development (R&D). China ranked second internationally in gross domestic expenditure on R&D in 2018, ranking first and second for such expenditure by government and higher education, respectively (UNESCO Institute for Statistics 2020). Such spending could result in higher use of research evidence in government. One example shows a strong role for scientific evidence-based decisions in relation to air pollution control in the Pearl River Delta (Zhong *et al*. 2013). The researchers argued that the success of interactions between policymakers and research institutions in this region and internationally were pivotal in regional air pollution reductions. However, there could be other reasons for China’s high R&D expenditure (such as enhancing prestige) and a high production of research evidence does not necessarily correlate with its use by policymakers.

Chinese governance is characterised by a centralised state with formal authority that sets strategic priorities through nationwide Five-Year Plans, which are implemented through targets and performance measures to lower tiers of government. Priorities in such plans:

may reflect the personal views of powerful leaders, the preferences of major interest groups, influential theories about economic development, assessments of what is needed to maintain the stability of the system, the ideas of prominent scientists, responses to rising public pressures, and so forth.

(Young *et al*. 2015: 165)

Official research institutions (*e*.*g*. Chinese Academy of Sciences) play an important policy advisory role, and are sometimes included under the term ‘think-tank’. They are not necessarily independent, but can be seen as:

stable and autonomous organisations that research and consult on policy issues to influence the policy process.

(Xufeng & Lan 2007: 453)

Similarly, ‘experts’ are not necessarily neutral because they are usually employed by:

government-sponsored think tanks or universities; consequently, their proposals must be in accordance with the doctrine of a political party.

(Xufeng 2013: 284)

Other modes of expert influence include ‘semi-official’ and ‘civilian’ think-tanks, in which the former benefit from pre-existing links to government, while the latter rely more on personal ties and expert knowledge to create impact (Xufeng 2009). Policies based on scientific expertise are likely to be pursued in China, but this expertise is generally within or closely linked to government institutions, a topic which requires further investigation.

This study responds to calls for investigation of local ‘cultures of evidence use’ in a specific policy context (Oliver & Boaz 2019), considering urban sustainability and health policies in two Chinese cities. The present research is part of a large transdisciplinary project called Complex Urban Systems for Sustainability and Health (CUSSH), which aims to work with six city partners (Nairobi, Kisumu, Beijing, Ningbo, London and Rennes) to ‘ensure wide participation in the development (“co-creation”) and use of research evidence by decision-makers’ (Davies *et al*. 2021).^1^ China is an important case for understanding environmental health decision-making given its status as the largest producer of CO_2_ emissions, comprising 28% of the global total (Friedlingstein *et al*. 2019) and its status as the most populous country in the world with 1.43 billion people in 2019, comprising 19% of the global total (United Nations Department of Economic and Social Affairs, Population Division 2019).

The aims and objectives of the present research are informed by the CUSSH programme theory (Moore *et al*. 2021), which sets out multiple assumptions regarding the value of evidence for policymakers to be tested through the research. The aim is to understand urban health and sustainability actors’ perspectives and influences on the use of scientific evidence in policy and decision-making. Specifically, this paper explores actors’ interpretation of ‘evidence’ as a concept, mechanisms to obtain different types of evidence for policymaking, existing collaborations between researchers and policymakers, and the perceived usefulness of evidence for policymaking. The research team includes civil servants and academics working across public health, epidemiology, urban planning, engineering, behavioural science and system dynamics. The research approach involved literature review, semi-structured interviews and thematic analysis.

## 2 China’s Governance for Urban Sustainability and Health

Political modernisation and increasing wealth in China led to environmental reforms and a degree of decentralisation of environmental decision-making (Kostka & Nahm 2017; Mol & Carter 2006). Local decision-making on environmental problems has had mixed results due to limited financial incentives and powerful economic interests that block reforms (Mol 2009; Mol & Carter 2006; Young *et al*. 2015). Despite recent Five-Year Plans coupling environmental and economic priorities, city competition has been characterised by ‘GDPism’ because local officials were measured and promoted on economic growth indicators (Wu 2016), resulting in uncoordinated urban development and the slow achievement of environmental goals (Vogel *et al*. 2010). Urbanisation by processes of agglomeration, or clusters of cities (Fang & Yu 2017), may have produced fragmented and overstretched local governance systems (Lu *et al*. 2019; Schneider & Mertes 2014), inhibiting effective environmental planning.

Central government plays an important role in setting both health and environmental agendas. President Xi Jinping announced a national ecological civilisation agenda in 2013, putting a comparatively greater emphasis on social and environmental over economic goals, in which health was seen as the ‘centrepiece of sustainable development in China’ (Yang *et al*. 2018: 1). The importance of health was articulated in the Healthy China 2030 plan (Central Committee of Chinese Communist Party & State Council 2016), described by Yang *et al*. (2018: 3) as a ‘dramatic departure from traditional strategies’ regarding health service improvement to a more holistic governance model covering environmental and social determinants of health through distributed health-in-all-policies approaches.

The Healthy China 2030 strategy explicitly links a healthy population with a healthy environment. It calls for increasing the public’s knowledge of the environment, enhanced environmental monitoring, a risk management system and increased public participation. In July 2019, the State Council issued guidelines for the actions and implementations of Healthy China 2030, and established the Promotion Committee of Healthy China, assembled by leaders from the State Council and the office under the National Health Commission. The committee focuses on monitoring and evaluation of Healthy China via indicators (National Health Commission 2019). The Healthy China 2030 plan is a key driver of environmental health policy, and one of its four principles is to be guided by science (Yang *et al*. 2018).

The 2014 revised national Environmental Protection Law was described by Zhang *et al*. (2015) as a potential ‘game changer’, produced through an ‘unprecedented’ and ‘highly controversial’ process that has achieved significant advancements to strengthen environmental policy, public participation and information disclosure. Instrumental in the revision process of this law was the Ministry of Ecology and Environment (MEE), formerly known as the Ministry of Environmental Protection. The ministry’s role is to implement and enforce environmental laws and regulations, and to provide support for policies relevant to environmental issues, such as Healthy China 2030 and the 13th Five-Year Plan Environment and Health Work (MEE 2020).

The dynamics of sustainable urbanisation policy in China illustrate the delicate power balance between central government strategy and local influence over outcomes ‘on the ground’ (Chung *et al*. 2018). Cities are the tier of Chinese government with primary responsibility for implementing national and local environmental regulations, and they are under pressure from both higher government tiers and the public (Zhang *et al*. 2018). Monitoring of environment and health policy implementation is part of central government’s strategy to ensure progress. In relation to environmental data, the central government has:

allowed and even actively stimulated information disclosure and media openness often with the idea of building countervailing power against local governments and powerful, local polluters.

(Zhang *et al*. 2017: 59)

In summary, existing research demonstrates that evidence plays an important role in central and local environmental governance systems in China. However, there is a lack of research on what Oliver & Boaz (2019: 6) call ‘cultures of evidence use’ in the overlapping policy areas of urban sustainability and health.

## 3 Methods

This study is based on a qualitative interview analysis. A total of 23 participants were interviewed in two Chinese (CUSSH partner) cities, Beijing (*n* = 12) and Ningbo (*n* = 11). Participants were purposively sampled by the Chinese research partners based on their professional networks to include those with knowledge of urban sustainability and health challenges and associated policy processes. Participants were recruited from the following types of organisations: municipal research and administration agencies, university departments, community service centres, and primary care services. Participants had professional expertise in the following areas: climate change; water, soil and air pollution; medicine, public health and epidemiology; urban planning; economic development; meteorology; and waste management.

Interviews took place in Beijing (20–21 May 2019) and Ningbo (23–24 May 2019): they were semi-structured, audio-recorded and transcribed. Translators were present and they were members of the research team to aid with reflexivity and interpretation (Temple & Edwards 2002; Wallin & Ahlstrom 2006). Details about the interview guide, translation and transcription process, and the thematic analysis process are reported in the supplemental data online, including reflections on conducting this analysis as a transdisciplinary research team, using the reporting criteria recommended by Pineo *et al*. (2021).

## 4 Results

An overview of the health and sustainability evidence and governance structure described by participants is followed by three themes regarding the role of government priorities, the public and barriers in evidence generation and use.

### 4.1 Environment and Health Evidence and Governance System

This section reports foundational information regarding participants’ descriptions of the overarching governance system for environmental health in China, the routes through which evidence informs governance and the types of evidence described by participants. The following sections will build on this foundation to explore specific themes related to evidence use.

#### 4.1.1 Governance structure

**Figure 1:**
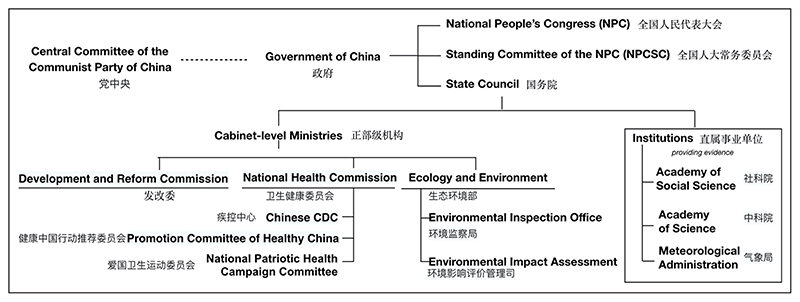
Central government’s decision-making structure for urban health and sustainability described by interview participants (note the structure is not comprehensive). Institutions provide evidence to ministries and other organisations and departments.

Participants described environmental health governance in China as consisting of a hierarchical decision-making structure whereby priorities are determined centrally and filter down to ministries and other tiers of government (Figure 1). A participant summed this up as:

It’s powerful. […] If the central government have a policy, the local have to follow.

Central government’s key role in governance for environmental health was linked to these issues being ‘more sensitive’ and therefore ‘Chinese government attaches great importance to it.’

Policy priorities are first articulated at the annual joint meeting of the National People’s Congress and National Committee of the Chinese People’s Consultative Conference. Priorities are then communicated to the State Council to investigate and issue specific guidelines, through the overarching and subject-specific Five-Year Plans, via the cabinet-level ministries and supporting institutions. This process was described as a channel to integrate public opinions with research evidence. A Beijing health expert noted:

they take some investigation to confirm if this is a real problem or not, then how to adjust their policy or their action plan to respond. To meet the concerns […].

Participants noted that specific issues proposed by representatives of the People’s Congress, at either national or local provincial levels, were taken seriously.

Participants described how the National Development and Reform Commission, a macroeconomic management agency, had considerable power in determining land-use policies impacting urban health and sustainability. The 13th Five-Year Plan (2016–20), issued by the Commission, included the Healthy China agenda in the Green Development section, calling for green and environmentally friendly industries, resource conservation and other measures. The other relevant cabinet-level ministries for environment and health governance include the National Health Commission, which oversees the Centres for Disease Control and Prevention and the Ministry of Ecology and Environment. The 13th Five-Year Environment and Health Plan, developed by this ministry, was noted as underpinning the ministry’s work because it contained key measures for environmental sanitation: ‘this is why our subsequent work has been strongly supported’.

The emergence and implementation of Healthy China 2030 was frequently referenced by interview participants. This national strategy has filtered down to impact the work of all environmental health (and even some healthcare) professionals, as one participant described:

In fact, it is not so easy to start, but the relative development in the past two years will be faster. In fact, the main reason is that the Chinese government attaches great importance to it. In fact, President Xi proposed a plan to build a beautiful China and ecological civilization, as well as a plan for a Healthy China 2030.

Interviews in Ningbo showed how the national agenda was implemented at the local level through the Healthy Ningbo 2030 project, described as a ‘government-led and social participation model [… that] has little to do with capital investment’. As part of a network of pilot projects across the country, Ningbo was required to set up a Leadership Committee of senior officials from city government departments, chaired by the mayor. The city aims to integrate health through all decision-making (*e*.*g*. through increased use of health-impact assessments) using existing departmental budgets to progress the project.

#### 4.1.2 Channels for evidence use by government

The provision of research evidence to cabinet-level ministries and other tiers of government occurred through several formal channels. Institutions, such as the Academy of Social Science, provide research evidence and deliver administrative functions for the ministries and departments (Figure 1). University experts can collaboratively produce evidence with or for local government agencies or research institutes. Interview participants had diverse perspectives on how their research evidence was used by government. Some agencies and institutions cooperated in a ‘service-oriented manner’ in response to government requests, *e*.*g*. by providing reports and data to one another. Such cooperation was described as ‘uni-connected, that is, there is a lack of a coordinated intermediate sector at the moment’.

Typically, experts from the scientific institutions (Figure 1) provided reports to the cabinet-level ministries and provincial/municipal government departments for consideration by officials. The central government research institutes have a remit to influence research strategies for different provinces: ‘all the idea is passing from the centre to the provincial and municipal’. Experts in these institutions were clear that their role did not involve policymaking or policy advice, and that the government approved topic areas for research. Such approval was seen to be influenced by government scientists’ data from (environmental and health) surveillance and expert opinion. When asked what policies a participant would like to see to improve air pollution, one municipal research institute expert responded: ‘No way. We cannot provide any policy support.’

Another participant was more hopeful to see policy change resulting from their study. They said that their study about air quality and health was very likely to inform policy. The method to achieve this impact involved demonstrating the strength of the research through publication in ‘a very high-level journal’ or an expert report for government. The participant said:

it’s produced by all the experts, they cannot, you know, they do not criticise your result. The government will believe; they will release some policies or even the laws to change the city policy.

In addition to formal reports and publications, one participant from a scientific institution described other routes through which their evidence informed government, such as briefings to local government officials including ‘township cadres […] the mayor [and] the secretary of the municipal party’. Briefings were seen as a key mechanism to disseminate knowledge about climate change and risk of extreme weather disasters, and this was also part of building a long-term relationship with officials for ongoing collaboration:

You tell [the senior person being briefed], in fact, that this reserve of knowledge may have some direct use. For example, if you give him information right away, he can assign work right away and then do it. On the other hand, it is a reservation and an improvement in personal achievement. Then he has some understanding of some of your problems. I think it may be on both levels.

An academic participant told of their experience of producing evidence for government. They found it difficult to influence government:

We’ve got a team. We are doing all these things. We get it published. But whether the government are going to appreciate that or not, we don’t know.

However, they felt that specific international events could drive government attention in a topic, resulting in increased research. For example, they said that six months before the 2016 G20 summit there was government interest in research:

So this province, Shanghai and the Jiangsu province, they put a lot of money together just to do these preventive measures to ensure the [air] quality for two days. […] So in Shanghai a lot of [research] teams are working on air quality.

#### 4.1.3 Types of evidence used in environmental health governance

Multiple types of information were referenced as sources of knowledge, or evidence, for environment and health governance, including routine monitoring, sampling of exposure data and modelling (Table 1). The majority of evidence was prepared for ‘risk factor monitoring and disease monitoring’. This section describes how participants viewed these types of evidence as informing policy and decision-making.

Routine monitoring and surveillance data (*e*.*g*. of environmental risks and health behaviours) were described as informing policy development and evaluating policy impact. Evidence was used to ‘prioritise’ air pollution control policies:

First is, coal, burning coal because it’s the most polluted. Second would be the vehicles. And they decided this sequence based on surveillance data from the environmental department.

Evidence was also seen as driving a full cycle of informing and evaluating policy. A nutrition expert said:

I think that every [nutrition] policy is based on the result of the surveillance. And also, the [nutrition] surveillance, the intervention and policy, and the surveillance again for that, that is the common cycle.

Modelling and forecasting data were described as inputs to policymaking, specifically in relation to air quality and meteorological studies. For instance, a modelling study about the urban heat island effect was provided to the urban planning department with the aim of influencing policy. It was unclear to the participant if this evidence would be used:

So we’ll tell them […] which part of our city has a more severe heat island effect. Then tell them about the target area and maybe they’ll think about it in the plan.

To summarise, participants said that priorities for urban health and sustainability were determined centrally and filtered down to cabinet-level ministries, scientific institutions, and provincial and municipal government via the Five-Year Plans. There were formal mechanisms for producing and using research evidence to implement the centrally determined overarching priorities. The types of evidence that participants had experience of producing or using in environmental health governance included routine monitoring, sampling of exposure data and modelling of exposures/health conditions.

### 4.2 Evidence Use and Government Priorities

There was frequent recognition that evidence generation and use was determined or affected by government priorities at multiple tiers. Participants described the government as being open to listening to experts, but not necessarily acting upon the information. They also noted that government support for a particular agenda opened opportunities for cross-departmental collaboration.

Central government set strategic policy directions that could be influenced by local-decision makers who made use of evidence. One example relates to the type of growth that would be permitted in the Yangtze and Pearl River deltas. Central government targeted these regions as major growth areas, but not in a way that would harm health:

And they will send out this requirement to say: ‘Now, we welcome investors, but there is no low-value added product activities and also, more importantly, there is no health unfriendly industries […] that was the central [government’s] idea.’

The form of growth was agreed with local decision-makers:

the seniors make the decision. It’s like the Jiashan city mayor, for example [and the] Development and Reform Commission.

The participant explained that these local agencies were increasingly drawing upon outside expert knowledge from an ‘external knowledge tank’ to inform their work. Such advice is procured through open bids:

They send out to the public and the scholars get asked from the universities. Also, we have some consulting firms. Anybody can apply to bid for these projects.

Here the policy agenda was set centrally with some local involvement, drawing upon research evidence.

Government was seen to evaluate the potential policy implications of research and the extent to which it matches their priorities. This evaluation influenced whether research findings were used in policy and decision-making. An environmental health expert thought that the government is:

probably more concerned about the economy [than the environment …] so I think that’s why some of our research results are also good but not well adopted by the Government.

A climate expert said that when research findings are presented to government, they ‘may feel what you suggest is right and integrate it to the work’. Alternatively, if they ‘think that you are unreasonable’ or ‘that what you have said is unreasonable’, then they will not use the research findings. In summarising their view, this participant said that they ‘believe the Government will still listen to the opinions and suggestions of experts’.

City government agencies described conducting programmatic work that was quite specific to the remit of their department and informed by local priorities. Climate adaptation and mitigation was described as a priority for Ningbo because it affected the public’s daily life, which may have supported cross-departmental evidence generation. In 2014, the city ‘suspended work and school in response to extreme weather’. Experts commented on a local climate feasibility study that resulted in city policies related to:

ventilation corridors in the city, to relieve the urban heat island effect and smog problems […] fresh air monitoring and network system construction, artificial rain strengthening, as well as low-carbon products […] according to local conditions.

Multiple city government departments contributed and used evidence, including the urban and transport planning departments.

### 4.3 Public Opinion Influencing Government Priorities

Participants frequently described how public opinion has directly influenced central government priorities and policy. Air pollution was commonly discussed in this regard, but also more specific environmental health incidents that received significant media exposure, public complaints and government reaction. Residents’ views were informed by their personal experiences (*e*.*g*. smelling or seeing air pollution) or media reports of specific problems that were investigated by environmental health experts. In this way, ‘evidence’ in the forms of public and technical knowledge could be inserted into government decision-making if it were elevated in importance by public complaints.

The process for the public to raise their views to government was described as flowing through formal channels via surveys, complaint hotlines, political consultation and the People’s Congress (Figure 1). One vector-borne disease expert said that the importance of this ‘bottom up […] from the local, the vulnerable populations and the local residents’ information has increased over time, but it has to be weighed against other types of information in a particular ‘balance’:

you have to consider the evidence but also you need to consider the complaints of the residents.

Environmental health evidence was gathered in response to public concerns and in turn this evidence informed policy. One environmental health expert described the government response to public complaints about a school built on contaminated soil, causing severe health effects for the pupils. This triggered an in-depth investigation by environmental health officials and widespread media coverage. The public were highly concerned because the ‘very high contamination levels’ affected children. As a result, the participant explained that:

The mind-set of those government officials [shifted], they’re going to pay much more attention when it comes to these public services […].

The participant also noted that some key environmental health issues do not receive media attention which creates challenges for raising public awareness and government attention.

The government was also perceived to be responsive to public concern regarding decision-making for extremely polluting industries. A controversial proposal to develop a xylene factory in a petrochemical industrial zone was overturned following public outcry: it was ‘the public that opposed it’. This occurred despite governmental approval, potential job creation and the developer’s promise to follow recommendations from an environmental impact assessment conducted by an international consulting firm. The developer tried to move the project to another city:

but now the media is so powerful, so the public over there have read such news which happened in Ningbo. […] Then it didn’t have the success anywhere in China.

The participant felt that the lack of trust between the public and government was a key factor in this scenario because the factory could have been developed in an environmentally friendly way, supporting a ‘win–win situation’. However, this has not been achieved because:

the trust between the public and the government is still very weak when it comes to these very heavy polluting industries.

Participants working in government confirmed that public opinion is important. In relation to poor water quality, one official said, ‘at the government level, we are more concerned about the reaction of the people’. The participant noted that public concern had led to policy that resulted in visible improvements. In turn, positive opinion about the water quality improvements further supported government policy in this area. In contrast, an ecology expert in Beijing noted that public participation is not commonly used to inform decision-making in their area of policy, partly because Beijing is a ‘special city’ (given its visibility internationally and in China) and because public opinion is not uniform:

Especially in Beijing, the decision-making is mainly led by the central Government, and the public participation is not enough, the effect may not be good. Because, after all, opinions are different. After all, Beijing is a special place, it is the capital, in short, the level of public participation in this project and other projects are relatively low.

Government priorities and public opinion were interrelated. Government priorities determine which topics are investigated by researchers, and the findings inform subsequent policies.

### 4.4 Barriers to Evidence Creation or Use

There were several barriers to environment and health evidence being created or used by policymakers. Participants suggested that the transparency of policy goals and reporting meant that policy targets were not based on evidence, but instead were determined by what could be achieved. Privacy considerations and lack of data-sharing across departments sometimes obstructed evidence generation. Some participants noted that the fast pace of government policy reduced the use of evidence to inform air pollution control policies.

Meeting published environmental targets such as those for pollution reduction was described as important. A participant said that:

if they publish this goal within the Five-Year Plan, they need to publish and measure the success.

One effect of this transparency was that policies should not be changed too frequently—‘you have to be very careful about it’—to avoid failing to meet the target. In another example, a participant noted that the policy target was carefully set to avoid failure: ‘you cannot set the threshold too high’. In these examples, participants described how the policy targets were not necessarily evidence based. Although targets were published and transparent, the process for determining these targets was ‘like a game’ and not widely known.

Transparency of goals and progress against them was notable across policy areas. Policy targets were described as being carefully monitored through environmental exposure data, although there was a perception that data manipulation had occurred in some cases. One participant noted that the local government agency had instituted a requirement for real-time public reporting of air quality data on a website to avoid any potential for data manipulation:

if they do it manually the data might have been manipulated. Because we know this data, the air quality is not that good, but from the data it shows very good. The reason why, actually the local officials working in that monitoring station they can manipulate data. So now all these things have been in a way blocked.

Although transparent public reporting was commonly referenced, data-sharing across government departments was a barrier for some public health activities. One environmental health expert had experience of monitoring air pollution, heatwaves and morbidity. There was cross-departmental collaboration to create a health warning system based on certain climatic conditions. There were some ‘data-sharing’ challenges:

because their weather data are as open to the public, but our health data are sometimes concerned about confidentiality.

However, they were able to cooperate to create the warning system. From their perspective, the value of the data was not necessarily to inform government policy but to support sensitive groups in the population to manage their own exposure:

They can make better use of our monitoring or research data and we can do some accurate education for them. […] I think this is also a meaningful thing.

Other barriers or restrictions to sharing data across departments were bureaucratic—‘lack of a well-coordinated department’—but also related to non-health departments not being supported to work outside of their remit, *i.e*. investigating issues pertaining to health and the environment.

Finally, the pace of change in air pollution control was also described as a barrier to research evidence influencing policy. An interview participant in Beijing described the undesirable consequences of banning coal-burning for low-income residents who suffered health risks from excess cold in their homes. However, it was difficult to influence government with this evidence, particularly when such evidence ‘challenges’ its policy or plan. In terms of air pollution control:

the government is quite strong and they’re anxious to change this situation very, very, fast […].

Thus, it was not possible to drive policy via evidence in that participant’s experience.

## 5 Discussion

This investigation found that scientific evidence was routinely used to inform environment and health governance priorities, policies and decisions. Evidence was primarily viewed as data produced by government agencies, whose priorities were centrally defined, while the use of academic evidence was less clear from this study. Public opinion was described as increasingly shaping environmental governance priorities; however, community participation in policymaking was generally limited to voicing opinions through formal channels.

Reflecting on the present findings of the ‘cultures of evidence use’ in China with findings in previous studies, a relatively large collection and use of environmental health data and a lower integration of diverse knowledge types in the Chinese cities can be noted. In order to consider the implications of these differences for China, including opportunities to strengthen governance capacities in this area, a framework for environmental governance by van der Molen (2018) is applied below.

### 5.1 Strengths and Limitations

The sample size (*n* = 23) of participants was relatively small, although the selection is based on well-informed and senior professionals working in government/scientific agencies. A clear repetition of the ideas discussed in the interviews indicated a point of saturation in terms of how evidence was used in the urban sustainability and health policy nexus. There were challenges with interpreting the meaning of the interview data, not only with regards language barriers but also understanding conflicting views. There are contradictory views in international academic and media discourse with coexisting accounts of China as a ‘reckless polluter’ and as ‘an emerging leader’ in global environmental mitigation (Zhang & Barr 2013: 6). Contrasting views were also reflected in the interviews, perhaps arising from the rapid pace of change in Chinese environmental governance and participants’ professional and individual experiences.

As a transdisciplinary and international research team, the positionalities of the authors (*e*.*g*. their personal characteristics, such as gender, ethnicity, class, nationality, *etc*., and plans for further research in these two Chinese cities) are likely to have influenced who participated, what they shared with the authors, and how those data were analysed and interpreted (England 1994). The authors’ Chinese collaborators were led by the senior co-investigator and team/colleagues at the Chinese Centres for Disease Control and Prevention (CCDC). The research team (including those who conducted interviews) comprised men and women with Chinese, American, Australian and European origins. The limitations of the study partly relate to the authors’ positionalities. First, participants may have had concerns about the intentions of the international researchers, and their responses may reflect these reservations and others. The authors sought to mitigate this limitation through the following actions: conducting the interviews primarily in Mandarin with the authors’ local partners; obtaining local ethical approval and communicating this in the participant information sheet; and ensuring complete anonymity for the participants. Second, the complex research topic and the diverse researchers and translators involved may have increased the risk that contextual knowledge from data collection was lost during data analysis (Mauthner & Doucet 2008) and likely had other influences on the interpretation of the results. To address this concern, the authors reconciled differences in their interpretations throughout the research process. There are strengths in the authors’ diverse team, with expertise in evidence use and urban governance working alongside Chinese health researchers, in terms of the study’s trustworthiness and authenticity, and the authors’ ability to challenge theoretical assumptions and encourage alternative interpretations to the data.

### 5.2 Capacities for Environment Governance in the Study Settings

It is evident from research and international statistics that in the last decade China has invested considerably in producing evidence and monitoring data to inform environmental governance (UNESCO Institute for Statistics 2020; Zhang *et al*. 2017). This section now discusses how this wealth of new data is being used to inform urban health and sustainability governance in two Chinese cities, and how the findings relate to previous studies in other contexts. A useful tool to guide interpretation of the findings is van der Molen’s (2018) framework of knowledge–governance relations. It conceptualises three forms of environmental governance capacities that are regulatory, adaptive and integrative. *Regulatory capacity* serves to ‘steer collective action with respect to the environment in desired directions’ and involves ‘normative goals or visions’ set out in regulations, policies or collaborative practices (van der Molen 2018: 20). *Adaptive capacity* is about understanding environmental change and using this knowledge to iteratively inform decision-making towards a desirable system state. *Integrative capacity* means to:

gain insight in diverging knowledges and normative perspectives and to bridge or integrate these for the sake of collaborative action.

(van der Molen 2018)

China’s centralised form of governance constitutes significant power that drives strong regulatory capacity. The participants described the process of setting regulatory targets or thresholds (*e*.*g*. for air pollution) as being opaque and driven by an aversion to failure. The central government controls what kinds of issues are researched and monitored and how the findings inform regulations, which may become more problematic in the longer term. The example of Beijing’s rapid decline in particulate matter since 2013 demonstrates the effectiveness of such centralised approaches (Wang *et al*. 2018), but there may be a plateau in pollution-reduction trends or hard-to-tackle industries that require other forms of governance capacities. Van der Molen (2018) suggests that such hierarchical and centralised models underpinning regulatory capacity should be complemented by adaptive and integrative capacities, which the present authors believe were less evident in Beijing and Ningbo.

The substantial environmental and health data collection described by participants could be a valuable resource for adaptive capacity in terms of environmental governance and specifically for emergency preparedness. The extensive and routine data collection and research observed in the present study permit government departments to set evidence-based policy, to adjust these policies over time and to make informed decisions during crises, be they extreme weather or new epidemics. According to van der Molen (2018), adaptive governance works by enabling learning about the effects of policies and adjusting them accordingly. In comparison with other academic studies (*e*.*g*. Pineo *et al*. 2019), the findings showed that in Beijing and Ningbo monitoring and surveillance (including data collection at small spatial scales) were more widespread and, perhaps most significantly, more systematically used to inform a cyclical policy process. This monitoring and policy cycle is rarely achieved elsewhere, underscoring the potential value of China’s approach. Given the predictions for increased crises caused by environmental degradation, the strengths in China’s research capacity may be a lesson for other nations. Emergency preparedness requires more than an understanding of risks and exposed populations; it also requires the scientific systems (researchers, protocols, *etc*.) and trusted relationships primed to respond to a crisis. This paper identifies an area of improvement in adaptive capacity related to data-sharing, which was found to be limited across departments and to the wider public, although this is changing (Zhang *et al*. 2017). Research on the use of urban health monitoring data in the US and Australia found that cross-sector and community access to such data were instrumental for achieving health-promoting environmental policy development and implementation (Pineo *et al*. 2020). In the context of complex urban sustainability and health challenges, China could increase its adaptive capacity by providing data to multiple government departments and other groups in society to enable whole-of-society approaches to framing problems and delivering solutions.

Despite the strength of China’s data systems, the findings indicate that the relative contribution of public knowledge as a source of evidence in urban sustainability and health governance, whilst increasing, could be further improved to increase integrative capacity. The revised 2014 Environmental Protection Law was a turning point for increased public participation in environmental governance (Zhang *et al*. 2017). The participants described cases where public opinion influenced policy- and decision-making, particularly in response to specific pollution threats. However, Beijing was described as a ‘special city’ in which public knowledge was not as influential in policymaking as national political objectives. This finding accords with a recent study by Zhang *et al*. (2020) that compares public participation in urban policy in Beijing and Guangzhou. They found that knowledge from experts and the public was influential in Guangzhou. However, in Beijing only expert knowledge was used in policy decision-making, which was strongly driven by national political goals.

Scholars have urged that diverse knowledge types are equally evaluated to inform health-promoting urban policies (Grant & Davis 2019) and environmental public health disasters (Généreux *et al*. 2019). Yet the inclusion of lay knowledge does not sit well with the hierarchical standards of evidence-based medicine. Even advocates of local knowledge note its limits in environmental health issues, partly because the general public may not be aware of the environmental problems that pose the greatest health risks (Corburn 2005; Zhang & Barr 2013). The necessity of finding a ‘balance’ between scientific and lay knowledge expressed by the study participants indicates that they were aware of this challenge, but some participants seemed to deprioritise such knowledge as ‘complaints’ that were not part of the ‘evidence’. Van der Molen (2018) highlights the risks of excluding diverse knowledge types in environmental governance, noting that integrating conflicting views is essential to enable collaborative action. As previously noted, progress on urban sustainability and health issues cannot be solely driven by regulatory capacity and the need for integrative capacity may increase over time as the public, industry or other groups push back at central directives. Multi-stakeholder deliberation platforms (Garard *et al*. 2018) are a method to balance diverging knowledge claims that may be appropriate in China, but this requires further investigation.

## 6 Conclusions

This study has highlighted the dynamic and diverse sustainability and health issues affecting Chinese cities, and the complex interconnections between national policy agendas, the public and evidence. Evidence-based policy is rarely linear and the clash of health evidence with economic objectives is a well-known challenge (Moore *et al*. 2021). City leaders in China, including those in the focus areas of Beijing and Ningbo, must manage the often-competing requirements of the public and other local actors, with the targets and mandates of state policies. Key strengths and areas for improvement are highlighted above for urban health and sustainability policymaking in the study settings using van der Molen’s (2018) framework.

China has many strengths in environmental governance that are lacking in other contexts, particularly regarding regulatory capacity and the extensive collection and use of monitoring data to inform and evaluate policy. However, there is a need to explore ways to improve integrative capacity, especially regarding the use of residents’ knowledge. Mechanisms for extending open-data protocols to health data (suitably aggregated and anonymised) would benefit from further investigation in order to increase the potential for cross-departmental collaboration and adaptive capacity. Strengthening these existing gaps could bolster the effectiveness of China’s environmental governance, leading to a responsiveness to environmental challenges that is lacking in other nations.

## Supplementary Material

Supplemental data on the methods, reflections on the research process and key government documents for the Healthy Cities 2030 agenda can be accessed at: https://doi.org/10.5334/bc.90.s1

Supplementary material

## Figures and Tables

**Table 1:** Sources of evidence described by participants with specific examples of data and evidence use.

Source of Evidence	Examples of Data	Example of Evidence Use
Routine monitoring and surveillance	Outdoor air quality (greenhouse gas inventory) Dioxin inventory Long-term meteorological or climate data Health conditions including dietary behaviour, hospital/general practitioner records	Informing prioritisation of policy measures Evaluation of policy or demonstration projects Public notifications of high environmental risks (*e*.*g*. air pollution)
Sampling	Food, water and soil sampled for toxins/bacteria Indoor air quality and radon monitoring (in a limited number of dwellings) Biological samples (blood, urine, nails and hair) tested for toxins	Health-protection activities of public health agencies (*e*.*g*. closing industries exceeding pollution levels or with contaminated food)
Modelling and forecasting	Air quality Urban heat island effect Climate feasibility assessment	Land use or energy policy Public notifications of extreme weather

## Data Availability

Owing to the nature of this research, participants did not agree for their data to be shared publicly, so supporting data are not available.

## Policy Relevance

- Insights are provided into the ‘cultures of evidence use’ in urban sustainability and health governance in two Chinese cities, noting the complex interconnections between national policy agendas, the public and evidence. Policies are centrally led and informed by officially commissioned scientific evidence.
- Environmental health data were used for monitoring policy implementation, and they were interpreted with careful consideration of public opinion and economic priorities.
- There were opportunities for local priorities to shift the policy agenda, particularly when the public became aware of a specific environmental health threat.
- Environmental governance could be strengthened in these settings through increased cross-sector data-sharing and integration of diverse knowledge types.
- A strength of China’s approach is routine data collection that feeds a monitoring and policy cycle rarely achieved elsewhere. This cycle provides feedback to government departments, allows policy adjustment over time and can inform decisions during crises.

## Footnotes

1
**Note**
For details about the CUSSH project, see https://www.ucl.ac.uk/complex-urban-systems/.
