# Supplementary material for: Evidence-informed urban health and sustainability governance in two Chinese cities
Source: Build Cities. Author manuscript; Available in PMC 2021 Nov 30. (PMC7612054; doi:10.5334/bc.90)
Supplement: Supplementary material [file EMS140019-supplement-Supplementary_material.pdf]

Pineo, H., Zhou, K., Niu, Y., Hale, J., Willan, C., Crane, M., Zimmermann, N., Michie, S., Liu, Q., and Davies, M. (2021). Evidence-informed urban health and sustainability governance in two Chinese cities. *Buildings and Cities*.
https://doi.org/10.5334/bc.90

Supplemental data

Contents

1. Methods 2
2. Reflections on the research process 4
3. Key government documents for Healthy China 2030 agenda 7
4. References 8

1. Methods

Participant recruitment was led by our senior co-investigator from Chinese Center for Disease Control and Prevention. He arranged participant invitations and interviews according to a pre-defined set of criteria about participants. We pre-defined the data collection and analysis protocols prior to recruiting participants. The data collection protocol stated that interview participants would include 'local policy makers (more and less senior), some academics, NGOs, representatives of community groups'. This mixture of participants was achieved in our sample, however we have not reported job titles to ensure anonymity of participants.

The interview guide (Table S1) was informed by our conceptual framework and agreed through discussion with the transdisciplinary research team. There were often multiple interviewers (1-2), translators (1-3) and participants (1-3) present for an interview. Interviews took place at Chinese Centres for Disease Control and Prevention offices in Beijing and Ningbo, or participants' offices.

Table S1: Semi-structured interview guide

	**Questions**
**1**	Can you start by telling me about your role in INSERT ORGANISATION?
**2**	Let's start by focusing our discussion on a recent typical major development/policy that you've been involved in. Can you talk me through the background to this project and your role in it?
**2a**	To what extent were urban sustainability and/or health considered in this project? Is that typical?
**3**	Who was involved in instigating this project – can you write down the organisations and stakeholders?
**3a**	Are there any other organisations that later became involved?
**4**	Can you tell me about your organisation's objectives or goals for the project?
**4a**	Do more important goals exist independent of this project for the development of the city as a whole?
**4b**	Why are the goals that you initially listed for the project important for your organisation?
**4c**	And what are the other organisations and stakeholder groups seeking to achieve?
**5**	What information helped your organisation arrive at these objectives/goals for the project?
**5a**	Did any organisations support or object to the proposed project and use some evidence/information to back up their position?
**6**	In relation to this project, how were decisions made to move from the initial goals to the current plans/policy?
**6a**	Who was involved in making decisions in this process?
**6b**	How is scientific evidence (research-based findings) used in informing these decisions?
**6c**	Do you think research findings can be used more effectively than this? If so how?
**7**	What barriers have made the achievement of these goals difficult recently or in the past?
**8**	What enablers, levers or facilitators have helped the achievement of these goals recently or in the past?
**9/10**	*Questions 9 to 10 related specifically to the CUSSH project and they were not analysed for this study.*
**11**	Thank you for your time. Is there anything else you would like to tell me about the topics we've discussed today?

Audio-recordings were transcribed in London using Chinese and English-speaking transcribers who provided additional translation if participants' words were not fully translated during interviews (Wallin and Ahlstrom, 2006). Researchers listened to the audio-recordings and checked for errors in written transcripts. We analysed interview data using thematic analysis (Braun and Clarke, 2006; Nowell *et al.*, 2017), adopting a hybrid inductive and deductive coding approach (Fereday and Muir-Cochrane, 2006), analysing data at latent and semantic levels. We derived a codebook using broad categories of policy processes (Table S3) from the conceptual framework. Each of the transcripts were coded in NVivo qualitative data analysis software (QSR International Pty Ltd., version 11.4.3, 2017) by two researchers.

In keeping with our conceptual framework, we have adopted theory-neutral or theory-crossing concepts to guide our exploration of the data. This was informed by a recent comparison of policy theory by Heikkila and Cairney (2017) and the literature review. The preliminary codebook in Table S2 defines categories for deductive data analysis. Inductive coding was used to explore the data within these broad themes in policy studies. We added two additional categories (governance and policy process) and multiple codes through inductive analysis. Studies on evidence use and policy in China informed our coding and interpretation of emerging results.

Table S2: Preliminary codebook of categories based on theory-neutral or theory-crossing concepts derived primarily from Heikkila and Cairney (2017)

**Category**	**Description**
Actors	Key people/positions involved in governance and/or policy-making
Institutions	'rules, norms, practices, and relationships that influence individual and collective behaviour' (Heikkila and Cairney, 2017, p.200).
Networks	'relationships between actors responsible for policy decisions and the "pressure participants" (Jordan, Halpin and Maloney, 2004), such as interest groups with which they consult and negotiate' (Heikkila and Cairney, 2017, p.200).
Ideas or knowledge	'…ways of thinking or the knowledge that plays a role in the policy process. … may include beliefs, knowledge, worldviews, and shared definitions of policy problems, images, and solutions within groups,

	organizations, networks, and political systems. Some ideas or beliefs may be taken for granted or rarely questioned—such as core beliefs, values, or paradigms. Others may be more malleable, such as proposed solutions to policy problems' (Heikkila and Cairney, 2017, p.200).
Policy context	'the wide array of features of the policymaking environment that can influence policy decisions. It can refer to the often-changing policy conditions that policymakers take into account when identifying problems and deciding how to address them, such as a political system's geography, biophysical and demographic profile, economy, and mass attitudes and behavior (Hofferbert 1974). It can also refer to a sense of policymaker "inheritance"—of laws, rules, institutions, and programs—on entry into office (Rose, 1990)' (Heikkila and Cairney, 2017, pp.200–201).
Events	'Events can be routine and anticipated, such as elections that produce limited change or introduce new actors with different ideas. Or they can be unanticipated incidents, including social or natural crises or major scientific breakthroughs and technological changes (Weible, 2017)... Their unpredictability makes them difficult to theorize, and they can often be treated as "errors" or external factors providing an additional source of explanation. Or they can be incorporated within theories that focus on how actors interpret and respond to events.' (Heikkila and Cairney, 2017, p.201)
Evidence	Evidence is a 'patchwork' of different types of information (Ingold and Monaghan, 2016, p.181). It may include academic research, trials, literature reviews, needs assessments, surveys of public views or preferences, public consultation, case studies, expert opinion, routine data and statistics (Lorenc *et al.*, 2014).

2. Reflections on the research process

Several procedures were followed to ensure rigour and build shared understanding across the research team. We reflect and report on this study using the reporting criteria recommended by Pineo et al. (2021) for transdisciplinary research in Table S3.

Table S3: Reflections from our study using Pineo et al. (2021) recommended reporting criteria for transdisciplinary research

Stage	Criteria	Approach taken in this study
(Pre-)Development	How was the project team formed?	For the CUSSH project, the project team formation is described by Davies et al. (2021). The team for this specific study emerged through conversations at a meeting in 2018 that included CUSSH partners and wider participants.
	Which organisations are included and what are their roles?	The main organisations in CUSSH are described by Davies et al (2021). This specific study includes academic partners from University College London (UK) and The University of Sydney (Australia, and the CUSSH partner in China at the Chinese Center for Disease Control and Prevention. Thus, we span academic and government agency organisations.
	How did partners agree on the problem or mission?	The idea to investigate the use of scientific evidence by decision-makers was a key objective of the CUSSH project (Davies et al., 2021, Moore et al. 2021)..
Co-learning	Who attended project meetings?	Project meetings for this specific study were attended by all of the co-authors or smaller groups of the co-authors, depending on individual availability and the meeting topic.
	What activities were used to create or share knowledge with diverse stakeholders?	The data collection and analysis procedures were agreed in shared documents by all co-authors. We used early discussions of our literature review to understand the Chinese context and policy research concepts.
	If relevant, how were conflicts resolved about the value of diverse knowledge types (e.g. technical knowledge, experiential knowledge, etc.)?	In this specific part of CUSSH we did not encounter conflicts about the value of diverse knowledge types. We agreed on the importance of investigating the use of evidence in policy-making through social research methods.
	How did the project build capacity within and beyond project partners?	Within the project, less experienced members of the project team were encouraged to learn new skills in qualitative data collection and analysis. They were mentored by other team members, including conducting interviews together, reflecting on the learning and then conducting interviews independently. We do not anticipate building capacity beyond our project partners with this specific study, except for through dissemination of the research process and outcomes.
	How did partners build and maintain trust throughout the project?	We took multiple opportunities to meet in-person (at CUSSH meetings and scientific conferences) and through regular video-conference meetings to develop trust. We discussed potentially sensitive components of this research and responded to those concerns through careful reporting and data management processes.

Stage	Criteria	Approach taken in this study
Reflection & Refinement	Which processes (or activities) were used to reflect upon the research and emerging results?	Emerging codes and themes were developed and reviewed iteratively to ensure clarity of interpretation across researchers (HP, KZ, CW), including analysing small portions of the data and then comparing and discussing results (Olesen et al., 1994).
	Which indicators were used to monitor progress/impact?	The wider CUSSH project includes evaluation indicators, see Moore et al. (2021).
	Who managed/participated in monitoring and reflection processes?	Reflection on this specific study was undertaken by all co-authors through shared research journals, meetings and commenting on the preliminary results. A parallel and more comprehensive process of reflection is embedded in the CUSSH programme theory (Moore et al. 2021).
Conceptualisation	How were diverse assumptions, theories and knowledge types integrated to form the project's research questions and approach?	We developed a protocol for this study in which we iteratively discussed and agreed our methodology. The protocol included an appendix in which we made our assumptions explicit about the use of evidence in policy-making. This manuscript reports one strand of analysis, but our protocol included other theories and codebooks to analyse the same dataset for different purposes.
	Which research governance processes were established and were these adjusted over time?	We used the CUSSH governance processes which included regular work package meetings and wider team meetings to discuss this work. We also held ad hoc meetings to plan data collection and conduct the analysis.
	Did the project create/adopt a conceptual framework and/or Theory of Change and how did this occur?	Our protocol included our conceptual framework for this specific study, which adopted theory-neutral or theory-crossing concepts about policy-making and evidence (see Table S2). We also referred to the wider CUSSH conceptual framework and theory of change (Moore et al. 2021).
	Which activities were used to exchange perspectives and knowledge when conceptualising the research approach and who was involved?	Within the wider CUSSH project there were two annual meetings that contributed to the conceptualisation of our research approach. In our specific study we used the process of writing and agreeing our protocol to exchange ideas.
Investigation	Which methods were used to gather and analyse data and how were these methods novel or integrative?	We do not believe that our data collection methods were novel or integrative. We adopted standard document review and semi-structured interview methods.
	How did the 'Investigation' stage build capacity within and beyond project partners?	We kept a shared research journal during the data collection and analysis period (HP, KZ, CW, JH), and used meetings to compare interpretations across the London and Beijing-based researchers (all co-authors).
	Who was involved in gathering and analysing data and what were their respective roles?	Data gathering was conducted by HP, YN, QL, KZ, and JH. . Analysis was led by HP with support from KZ, CW and JH. All co-authors were involved in discussing preliminary results, which led to further analysis. We were reflective about whether contextual knowledge was lost between the interview and transcript (Mauthner and Doucet, 2008) or during the translation (all co-authors).

Stage	Criteria	Approach taken in this study
Implementation	How was (new) knowledge from this project used to solve problems and or improve health? Or how do partners anticipate such change occurring?	We believe it is too early to say how this specific part of the CUSSH project will change urban health and sustainability evidence collection or policy-making.
	Which factors were required to make this change happen (e.g. funding or political will) and how were these achieved?	It is our goal to achieve change through our collaborative work with researchers and policy-makers across the CUSSH project. Our overall theory of change for the project is described in Moore et al. (2021).

3. Key government documents for Healthy China 2030 agenda

Table S4 shows a set of documents that are key to the delivery of Healthy China 2030 agenda. These documents were referenced by interview participants or found in our literature search.

Table S4: Key documents for the delivery of Healthy China 2030 agenda

Title	Publication Year	Publisher	Description
Healthy China 2030 strategy	2015	Central Committee of the Communist Party of China	President Xi initially proposed the Healthy China 2030 strategy in 2015, and then again in 2017 at the 19th National People's Congress.
Healthy China 2030 Planning Guideline	2016	State Council	The strategic goals of Healthy China 2030 were advanced by the State Council through specific planning guidelines. Then in 2019 the Health China guidelines were elaborated with a detailed assessment plan specifying evaluation criterion by the State Council.
Opinions of the State Council on Implementing the Healthy China Action	2019		
13th Five Year Plan Outline 2016-2030	2016	National Development and Reform Commission	The Five-Year-Plan is updated every five years. The 13th FYP (2016-2020), issued by the National Development and Reform Commission, included the Healthy China agenda in the Green Development section, calling for green and environmentally friendly industries, resource conservation, and other measures.
Establishment of the Promotion Committee of Healthy China	2019	State Council	To implement the strategy, in 2019 the Promotion Committee of Healthy China was formed to work across multiple tiers of government. The committee is responsible for implementation, monitoring and assessment related work of Healthy China.
Work plan to implementation of the Healthy China Action 2020	2020	Promotion Committee of Healthy China	The promotion committee's work plan to implement the Healthy China work in 2020.

4. References

Braun, V. and Clarke, V. (2006) Using thematic analysis in psychology. *Qualitative Research in Psychology*. 3 (2), pp. 77–101. doi:10.1191/1478088706qp063oa.

Davies, M., Belesova, K., Crane, M., Hale, J., Haines, A., Hutchinson, E., Kiesewetter, G., Mberu, B., Mojaheri, P.R.N., Michie, S., Milner, J., Moore, G., Osrin, D., Pineo, H., Pluchinotta, I., Prasad, A., Salvia, G., Tsoulou, I., Symonds, P., … Wilkinson, P., 2021. The CUSSH programme: learning how to support cities' transformational change towards health and sustainability [version 1; peer review: awaiting peer review]. Wellcome Open Res 2021, 6:100 (https://doi.org/10.12688/wellcomeopenres.16678.1).

Fereday, J. and Muir-Cochrane, E. (2006) Demonstrating Rigor Using Thematic Analysis: A Hybrid Approach of Inductive and Deductive Coding and Theme Development. *International Journal of Qualitative Methods*. 5 (1), pp. 80–92. doi:10.1177/160940690600500107.

Heikkila, T. and Cairney, P. (2017) Comparison of Theories of the Policy Process. In: Christopher M. Weible and Paul A. Sabatier (eds.). *Theories of the policy process* Fourth edition. [online]. New York, NY: Westview Press. Available from: http://search.ebscohost.com/login.aspx?direct=true&AuthType=ip,shib&db=nlebk&AN=1451128&site=ehost-live&scope=site [Accessed 11 July 2019].

Ingold, J. and Monaghan, M. (2016) Evidence translation: an exploration of policy makers' use of evidence. *Policy & Politics*. 44 (2), pp. 171–190. doi:10.1332/147084414X13988707323088.

Jordan, G., Halpin, D. and Maloney, W. (2004) Defining Interests: Disambiguation and the Need for New Distinctions? *The British Journal of Politics and International Relations*. 6 (2), pp. 195–212. doi:10.1111/j.1467-856X.2004.00134.x.

Lorenc, T., Tyner, E.F., Petticrew, M., Duffy, S., Martineau, F.P., Phillips, G. and Lock, K. (2014) Cultures of evidence across policy sectors: systematic review of qualitative evidence. *The European Journal of Public Health*. 24 (6), pp. 1041–1047. doi:10.1093/eurpub/cku038.

Mauthner, N.S. and Doucet, A. (2008) 'Knowledge Once Divided Can Be Hard to Put Together Again': An Epistemological Critique of Collaborative and Team-Based Research Practices. *Sociology*. 42 (5), pp. 971–985.

Moore, G., Michie, S., Anderson, J., Belesova, K., Crane, M., Deloly, C., Dimitroulopoulou, S., Gitau, H., Hale, J., Lloyd, S.J., Mberu, B., Muindi, K., Niu, Y., Pineo, H., Pluchinotta, I., Prasad, A., Roue-Le Gall, A., Shrubsole, C., Turcu, C., ... Osrin, D. Developing a programme theory for a transdisciplinary research collaboration: Complex Urban Systems for Sustainability and Health [version 1; peer review: 1 approved]. Wellcome Open Res 2021, 6:35 (https://doi.org/10.12688/wellcomeopenres.16542.1)

National Development and Reform Commission (2016). 13th Five Year Plan Outline. Available from: https://www.ndrc.gov.cn/fggz/fzzlgh/gjfzgh/201603/P020191104614882474091.pdf [Accessed 12 Nov 2020]

Nowell, L.S., Norris, J.M., White, D.E. and Moules, N.J. (2017) Thematic Analysis: Striving to Meet the Trustworthiness Criteria. *International Journal of Qualitative Methods*. 16 (1), pp. 1–11. doi:10.1177/1609406917733847.

Olesen, V., Droes, N., Hatton, D., Chico, N. and Schatzman, L. (1994) Analyzing together: recollections of a team approach. In: Alan Bryman and Robert G. Burgess (eds.). *Analyzing qualitative data* [online]. Abingdon, Oxon, London: Routledge. pp. 111–128. Available from: https://www.taylorfrancis.com/books/9780203413081 [Accessed 24 July 2020].

Pineo, H., Glonti, K., Rutter, H., Zimmermann, N., Wilkinson, P. and Davies, M. (2018) Urban Health Indicator Tools of the Physical Environment: a Systematic Review. *Journal of Urban Health*. 95 (5), pp. 613–646. doi:10.1007/s11524-018-0228-8.

Pineo, H., Glonti, K., Rutter, H., Zimmermann, N., Wilkinson, P. and Davies, M. (2019) Use of Urban Health Indicator Tools by Built Environment Policy- and Decision-Makers: a Systematic Review and Narrative Synthesis. *Journal of Urban Health* [online]. Available from: https://doi.org/10.1007/s11524-019-00378-wdoi:10.1007/s11524-019-00378-w.

Pineo, H., Turnbull, E.R., Davies, M., Rowson, M., Hayward, A.C., Hart, G., Johnson, A.M. and Aldridge, R.W. (2021) A new transdisciplinary research model to investigate and improve the health of the public. *Health Promotion International*. doi:10.1093/heapro/daaa125.

Promotion Committee of Healthy China (2020). Work plan to implementation of the Healthy China Action 2020. Available from: http://www.nhc.gov.cn/guihuaxxs/s3585u/202009/4757048a304b45e49d8073b33df0647d.shtml [Accessed 12 Nov 2020]

Rose, R. (1990) Inheritance Before Choice in Public policy. *Journal of Theoretical Politics*. 2 (3), pp. 263–291. doi:10.1177/0951692890002003002.

State Council (2016). Healthy China 2030 Planning Guideline. Available from: http://www.gov.cn/zhengce/2016-10/25/content_5124174.htm [Accessed 12 Nov 2020]

State Council (2019). Opinions of the State Council on Implementing the Healthy China Action. Available from: http://www.gov.cn/zhengce/content/2019-07/15/content_5409492.htm [Accessed 12 Nov 2020]

State Council (2019). Establishment of the Promotion Committee of Healthy China. Available from: http://www.gov.cn/zhengce/content/2019-07/15/content_5409494.htm [Accessed 12 Nov 2020]

Wallin, A.-M. and Ahlstrom, G. (2006) Cross-cultural interview studies using interpreters: systematic literature review. *Journal of Advanced Nursing*. 55 (6), pp. 723–735. doi:10.1111/j.1365-2648.2006.03963.x.

Weible, C.M. (2017) Introduction: The Scope and Focus of Policy Process Research and Theory. In: Christopher M. Weible and Paul A. Sabatier (eds.). *Theories of the policy process* Fourth edition. New York, NY: Westview Press.
